# HtrA4 is up-regulated during trophoblast syncytialization and BeWo cells fail to syncytialize without HtrA4

**DOI:** 10.1038/s41598-021-93520-1

**Published:** 2021-07-13

**Authors:** Mary Mansilla, Yao Wang, Rebecca Lim, Kirsten Palmer, Guiying Nie

**Affiliations:** 1 Implantation and Placental Development Laboratory, Centre for Reproductive Health, Hudson Institute of Medical Research, Clayton, VIC 3168 Australia; 2 Department of Molecular and Translational Science, Monash University, Clayton, VIC 3168 Australia; 3 Implantation and Pregnancy Research Laboratory, School of Health and Biomedical Sciences, RMIT University, Bundoora, VIC 3083 Australia; 4 Department of Obstetrics and Gynaecology, Monash University, Clayton, VIC 3168 Australia; 5 Australian Regenerative Medicine Institute, Monash University, Clayton, VIC 3800 Australia

**Keywords:** CRISPR-Cas9 genome editing, Reproductive biology

## Abstract

The outer layer of the human placenta comprises syncytiotrophoblast, which forms through fusion of cytotrophoblasts (syncytialization), and plays a critical role in maternal–fetal communication including nutrient/oxygen transportation and hormone secretion. Impairment in syncytialization inevitably affects pregnancy outcomes. High temperature requirement factor A 4 (HtrA4) is a placental-specific protease, expressed by various trophoblasts including syncytiotrophoblast, and significantly elevated in preeclampsia at disease presentation. However, it is unknown whether HtrA4 is important for syncytialization. Here we first examined HtrA4 expression in primary human cytotrophoblasts during syncytialization which occurs spontaneously in culture, and in BeWo cells which syncytialize upon forskolin stimulation. The success of syncytialization in each model was confirmed by significant up-regulation/secretion of β-hCG, and the concurrent down-regulation of E-cadherin. In both models, HtrA4 mRNA and protein increased concomitantly with syncytialization. Furthermore, the secreted levels of β-hCG and HtrA4 correlated significantly and positively in both models. We next knocked out HtrA4 in BeWo by CRISPR/Cas9. Upon forskolin treatment, control BeWo profoundly up-regulated β-hCG and syncytin-1, down-regulated E-cadherin, and at the same time increased the formation of multinucleated cells, whereas BeWo cells without HtrA4 did not alter any of these parameters. Our data thus suggest that HtrA4 plays an essential role in syncytialization.

## Introduction

The placenta, a pregnancy-specific organ, connects the developing fetus to the mother, modulates the intrauterine development, and adapts to changes in pregnancy to support fetal growth^1^. The outermost layer of the placenta comprises multinucleated cells called syncytiotrophoblast, which is formed via a process termed syncytialization whereby the underlying cytotrophoblasts undergo cell fusion. The syncytiotrophoblast layer forms a physical barrier that is in direct contact with maternal blood. It facilitates maternal–fetal communication via the production and secretion of pregnancy hormones and other factors, and via the transportation of oxygen, nutrients and waste materials^2,3^. These processes are essential for sustaining an adequate nutrient supply to the fetus^4,5^.

Impairment in syncytialization and abnormal syncytiotrophoblast function have been linked to premature birth, developmental defects and pregnancy complications^6–8^. Previous studies have reported that disruptions in syncytialization lead to the release of abnormal levels of syncytiotrophoblast-derived proteins, which enter the maternal circulation and may contribute to the development of pregnancy-related disorders such as preeclampsia^9–12^. However, more studies are needed to understand the basic biology of syncytialization in the human placenta.

A number of factors, such as human chorionic gonadotropin (hCG), glial cell missing transcription factor 1 (GCM1), syncytin-1 and E-cadherin, have been reported to play an essential role in syncytialization. Higher levels of β-hCG, GCM1, syncytin-1 and lower levels of E-cadherin are expressed by syncytiotrophoblasts compared to cytotrophoblasts. Therefore, changes in the expression of these genes are widely used as biomarkers of syncytialization. HCG is a heterodimeric glycoprotein composed of an α- and β- subunit, with β-subunit being specifically produced and secreted by cytotrophoblasts and multinucleated syncytiotrophoblasts^4^. During syncytialization, cyclic adenosine monophosphate (cAMP) induces phosphorylation of protein kinase A, which stimulates transcription factor GCM1^13^. Human GCM1, primarily expressed in the placenta, regulates the expression of the membrane fusion protein syncytin-1 and activates syncytiotrophoblast-specific hormones such as placental growth factor^14^. A positive feedback loop governs GCM1 activity where hCG expression elevates cAMP levels and activates GCM1. GCM1 then targets genes, such as syncytin-1, and the increased production of hCG promotes placental cell differentiation and fusion^13^. In contrast, E-cadherin, a transmembrane protein expressed in cytotrophoblasts that promotes cell–cell interactions, is down-regulated during syncytialization^15,16^. Abnormal expression of these syncytial markers has been shown to impair syncytialization and disrupt placental development and function, resulting in fetal growth restriction and pregnancy complications^12,17–19^. However, key molecular mechanisms underlying trophoblast syncytialization are far from being completely understood.

HtrA4 is the newest member of the HtrA family of serine proteases. Unlike other HtrA family members, which are widely expressed with functions linked to an array of biological processes such as cell growth, apoptosis, and cancer^20^, HtrA4 is highly unique because its expression is restricted to the human placenta^21^. HtrA4 is secreted by the placenta into the maternal circulation and its serum levels increase during early pregnancy before plateauing in the third trimester^21^. To date, we and others have reported that placental expression of HtrA4 is significantly increased in preeclampsia, especially in the early-onset subtype at the time of disease presentation compared to normal pregnancies^21–23^. Likewise, HtrA4 protein in the maternal serum is significantly elevated in early-onset preeclampsia^21–23^. Our studies further suggest that elevated circulating HtrA4 can adversely affect the integrity and function of maternal vascular endothelium and may play a role in the pathogenesis of preeclampsia^24–28^.

However, to date, the normal function of HtrA4 in the human placenta is unknown. Since HtrA4 is expressed in cytotrophoblasts and syncytiotrophoblasts^22,23^, this study aimed to investigate the functional role of HtrA4 in trophoblast syncytialization.

## Materials and methods

### Placental tissue collection

Term human placentas were collected from pregnancies immediately after elective caesarean section at Monash Medical Centre (Clayton, Australia) with the approval of the Monash Health Human Research Ethics committee (HREC reference number: 13357B). Placentas from the following pregnancies were excluded: underlying maternal disorders including preeclampsia, maternal hypertension, gestational diabetes, type 1 and type 2 diabetes, chromosomal abnormalities, advanced maternal age, BMI > 30, pregnancies with multiple fetuses, and suspected intrauterine infection. Tissues were processed within one hour of placental collection and in accordance with the relevant guidelines and regulations. Written informed consent was received from all participants.

### Primary cytotrophoblast isolation and purification

Placental villi were scraped from cotyledons that were rinsed 3 times with iced 1 × Hanks' Balanced Salt Solution (HBSS). Approximately 20 g of villi were subjected to 3 cycles of digestion in 25 ml of digest buffer [DMEM low glucose medium (Thermo Fisher Scientific, Scoresby, Australia), 1% penicillin–streptomycin (PS, Thermo Fisher Scientific), 0.25% trypsin (Thermo Fisher Scientific), 0.25% grade II dispase (Thermo Fisher Scientific), 0.1 mg/ml DNase I (Roche Molecular, Mannheim, Germany)] in a 37 °C shaking water bath for 15 min. The supernatant was carefully removed and filtered through a 100 μM cell strainer (Greiner Bio-One, Kremsmünster, Austria) into a 50 ml falcon tube. The supernatants were pooled and centrifuged at 350 g at 4 °C for 5 min. The cell pellet was then resuspended in 2 ml DMEM low glucose medium with 1% PS and 10% fetal bovine serum (FBS; Bovogen Biologicals, Keilor East, Australia), and loaded onto a Percoll gradient (70–5%, prepared in 50 ml tubes using 1 × HBSS). Samples were centrifuged at 1200 g for 20 min without brake applied. Cells in the layer between 15–27 ml (45–25% Percoll) were collected, washed with DMEM low glucose medium containing 1% PS, and centrifuged at 350 g for 10 min. The cell pellet was resuspended in DMEM/Ham’s F-12 medium containing 10% FBS, 2 mM L-glutamine (Sigma-Aldrich, Missouri, USA) and 1% antibiotic–antimycotic (Thermo Fisher Scientific).

To immuno-purify the isolated cells, 1 μg of mouse anti-human CD9 antibody (R&D Systems, Minneapolis, USA) was added per 1 × 10^7^ of cells and the mixture was rotated at 4 °C for 10 min. The cell suspension was then washed with 0.1% bovine serum albumin (BSA) in 1 × Dulbecco’s phosphate-buffered saline (DPBS) and centrifuged at 1000 g for 5 min. Cells were resuspended in 0.1% BSA/PBS (1 × 10^7^ cells/ml), mixed with goat anti-mouse dynabeads (Invitrogen, Carlsbad, USA) (50 μl per 1 ml of cells), then rotated for 30 min at 4 °C to allow binding. Dynabeads were pulled out using a magnarack (Life Technologies, Carlsbad, USA), the unbound cells were collected, and resuspended in DMEM/Ham’s F-12 medium containing 10% FBS, 2 mM L-glutamine and 1% antibiotic–antimycotic.

### Cell culture

Primary term cytotrophoblasts isolated above were seeded in 24-well plates (1.75 × 10^6^ cells per well) and cultured overnight in an 8% O_2_ incubator at 37 °C in DMEM/Ham’s F-12 medium containing 10% FBS, 2 mM L-glutamine and 1% antibiotic–antimycotic. The following day, cells were gently washed with DPBS to remove unattached cells, then replenished with fresh medium, and cultured for up to 72 h. Culture was terminated at 24 h, 48 h or 72 h respectively, and the conditioned media and cells were collected.

The human choriocarcinoma cell line BeWo (CCL98, American Type Culture Collection, MD, USA) was seeded in 6-well plates (0.5 × 10^6^ cells per well) and cultured overnight in a 5% CO_2_ incubator in DMEM/Ham’s F-12 medium containing 10% FBS, 2 mM L-glutamine and 1% antibiotic–antimycotic. The following day, cells were gently washed with 1 × DPBS to remove unattached cells, replenished with fresh DMEM/Ham’s F-12 media containing 2 mM L-glutamine, 1% antibiotic–antimycotic, 1% insulin/transferrin/selenium (ITS, Sigma-Aldrich), 1% BSA and 4.7 μg/ml linoleic acid (Sigma-Aldrich) containing either vehicle control (DMSO) or 20 μM forskolin, and cultured for up to 48 h. Culture was terminated at 24 or 48 h, and the conditioned media and cells were collected.

### Knockout of HtrA4 in BeWo

The HtrA4 gene in BeWo was knocked out by stable transfection with HtrA4 CRISPR/Cas9 KO Double Nickase Plasmids (Santa Cruz, Texas, USA), using Lipofectamine 2000 (Invitrogen, CA, USA) according to the manufacturer’s instructions. A vector only plasmid was transfected similarly as the control. Following transfection, cells were cultured in 20 cm dishes (2000 cells per dish) in DMEM/Ham’s F-12 media containing 10% FBS, 2 mM L-glutamine, 1% antibiotic–antimycotic, and 1 μg/ml puromycin (Sigma-Aldrich). Media were changed every 3–4 days until individual puromycin-resistant colonies formed. The colonies were transferred into a 96-well plate and eventually upscaled into T75 cm flasks. HtrA4 expression was assessed by RT-PCR and Western blot as described below. A HtrA4 knockout clone expressing negligible levels of HtrA4 (HtrA4-KO), and a control clone expressing HtrA4 similar to the un-transfected BeWo (Control), were selected and cultured in the presence of puromycin to study syncytialization using protocols described above for the wildtype BeWo cells.

### Total RNA extraction and reverse transcription

Total RNA was extracted using TRI Reagent (Sigma-Aldrich) as per manufacturer’s instructions. Contaminating DNA was removed using Turbo DNA-free kit (Ambion, Austin, USA). The RNA quality and quantity were determined by Nanodrop ND-1000 (Thermo Fisher Scientific). Complementary DNA (cDNA) was synthesized from 1 μg RNA using SuperScript III First-Strand kit (Thermo Fisher Scientific) as per manufacturer’s protocol.

### Real-time RT-PCR

Real-time RT-PCR analysis was performed as previously reported^25^, using a 7900HT Fast Real-Time PCR System (Applied Biosystems, USA) with the following conditions: 1) 95 °C for 10 min for enzyme activation, 2) 45 cycles of denaturation (15 s at 95 °C), annealing (5 s at 58 °C), extension (8 s at 72 °C), and a single fluorescence measurement at 76 °C for quantitation, and 3) dissociation curve assessment between 60 °C and 95 °C with continuous fluorescence measurement. All cDNA samples were diluted 1:80 and PCR reaction was prepared to a final volume of 10 µl with 5 µl SYBR Green PCR Master Mix (Thermo Fisher Scientific), 4 µl diluted cDNA and 1 µM final concentration of forward and reverse primers. The study used the following primers: 18S, 5’-CGGCTACCACATCCAAGGAA-3’ and 5’-GCTGGAATTACCGCGGCT-3’ (186 bp); β-hCG (*CGB*), 5’-CCCCTTGACCTGTGATGACC-3’ and 5’-TATTGTGGGAGGATCGGGGT-3’ (120 bp); syncytin-1 (*ERVW-1*), 5’-CCCCATCGTATAGGAGTCTT-3’ and 5’-CCCCATCAGACATACCAGTT-3’ (207 bp); E-cadherin (*CDH1*), 5’-GAAGGTGACAGAGCCTCTGGAT-3’ and 5’-GATCGGTTACCGTGATCAAAATC-3’ (122 bp); HtrA4 (*HTRA4*), 5’-GTCAGCACCAAACAGCG-3’ and 5’-GGAGATTCCATCAGTCACC-3’ (168 bp). Gene expression levels were normalised to 18S, and fold changes were calculated using ΔΔCt. All samples were run in triplicates, and experiments were repeated independently at least 3 times.

### Block RT-PCR analysis to confirm HtrA4 knockout in BeWo cells

PCR reaction contained 13 μl Go Tag green master mix (Promega, Madison, USA), 0.2 μM forward and reverse primers and 1 μL cDNA, with the total volume adjusted to 25 μl with ultrapure H_2_O. PCR reaction was run on Stratagene RoboCycler Gradient 96 Thermal Cycler machine (Aligent Technologies, Santa Clara, California) with the following cycling conditions: (1) 95 °C for 5 min, (2) 30 cycles at 58 °C for 45 s, (3) 72 °C for 10 min, and (4) 20 °C for 5 min. The PCR products were analysed on a 1.2% agarose gel (Promega) and stained with SYBR Safe DNA gel (Invitrogen). Images were taken with the Gel Doc EZ Imaging System (Bio-Rad Laboratories, Hercules, USA) and analysed using Image Lab Software (Bio-Rad Laboratories).

### Analysis of secreted β-hCG

Secreted β-hCG in the conditioned medium was quantified using the Beckman Coulter Unicel DXI 800 Immunoassay (Beckman Coulter, Miami, USA) at Monash Pathology, Monash Medical Centre (Clayton, Australia).

### Western blot analysis

Western blot analysis was performed as previously reported^27^. In brief, total proteins from cells were extracted using ice-cold lysis buffer containing 150 mM NaCl, 20 mM Tris-base (pH 7.4), 1 mM EDTA, 1% Triton X-100 (Sigma-Aldrich), and 0.1% Protease Inhibitor Cocktail Set III (Merck Millipore, Burlington, USA), and protein concentrations were determined by Bicinchoninic acid assay (Bio-Rad Laboratories). Equal amounts of total proteins from cell lysates (100 μg) or equal volumes of conditioned medium (40 μl) were separated on 10% Polyacrylamide SDS-PAGE gels and transferred onto PVDF membranes (GE Healthcare, Chicago, USA). Equal protein loading was confirmed with Ponceau staining (Ponceau S solution, Sigma-Aldrich). The membranes were then blocked in a blocking buffer [5% (w/v) skimmed milk in TBS and 0.2% (v/v) Tween 20 (TBS-T)] for 1 h at room temperature, and incubated overnight at 4 °C with primary antibodies (diluted in TBS-T), which included an anti-E-cadherin mouse monoclonal antibody (Thermo Fisher Scientific, 1:100 dilution), or an anti-HtrA4 mouse antibody (made in house, 1:1000). To further validate the selected HtrA4-KO and control cells, Western blot analysis was also performed with two additional HtrA4 antibodies (ab65914, 1:500 dilution, Abcam, Cambridge, United Kingdom; H00203100-B01P, 1:1000 dilution, Abnova, Taipei, Taiwan).

Following incubation with primary antibodies, membranes were incubated with a horseradish peroxidase (HRP)-conjugated goat anti-mouse IgG (1:2000, Dako, Glostrup, Denmark) or goat-anti-rabbit IgG (1:2000, Dako) for 1 h at room temperature, washed 3 times with TBS-T, then incubated with Lumi-Light Western Blotting Substrate (Roche Molecular). The bands were visualized using the ChemiDoc MP Imaging System (Bio-Rad Laboratories). To check loading control, the membranes were subsequently probed with an HRP–conjugated β‐actin antibody (1:2000; Cell Signalling Technology, Danvers, USA) at room temperature for 30 min. Protein band intensity was quantified using ImageJ software v1.8.0_172 (National Institute of Health, Maryland, USA). The experiments were repeated independently 3–4 times.

### Immunofluorescence and cell fusion quantification

Immunofluorescence staining of BeWo cells was adapted from a previously reported method^25^. BeWo cells were seeded on 22 mm glass coverslips (Thermo Fisher Scientific) that were placed in the 6-well plates, and treated with either vehicle control (DMSO) or 20 μM FK for 48 h. Cells were fixed with 4% paraformaldehyde (VWR, Radnor, USA) for 10 min and permeabilised with 0.1% Triton X-100 (Sigma-Aldrich) in PBS for 5 min. They were then blocked with 1% BSA in PBS for 2 h, and incubated at 4 °C overnight with the E-cadherin mouse monoclonal antibody described above (1:50 dilution). All procedures from here onwards were carried out in the dark at room temperature. The cells were washed three times with PBS, and incubated with a goat anti-mouse Alexa Flour 488 antibody (Life Technologies, 1:200 dilution) for 2 h. Nuclei were stained with 5 µg/ml 4′,6-diamidino-2-phenylindole dihydrochloride (DAPI; Sigma-Aldrich) for 10 min, and the coverslips were mounted onto glass slides with fluorescent mounting media (Dako). Images were taken at 60 × magnification using an Olympus DP70 camera and Olympus CellSens Standard Imaging Software v1.16 (Olympus, Notting Hill, Australia). Three to four images per treatment were taken randomly, and the entire experiments were repeated independently at least three times. To quantify cell fusion, the number of nuclei presented within single-nucleated vs multinucleated cells (containing two or more nuclei) were counted on each image, and fusion index was calculated as (number of nuclei within multinucleated/total nuclei) × 100, as previously reported^15^.

### Statistical analysis

Statistical analyses were conducted using GraphPad Prism 9.0.0 (GraphPad Software Inc., San Diego, CA, USA, www.graphpad.com). Data are expressed as mean ± standard deviation (SD), and comparisons were made using t-test or one-way ANOVA followed by Tukey’s post-hoc test, as statistically appropriate. Pearson correlation coefficient, expressed as ‘r’, was used to evaluate the correlation of the secreted levels of β-hCG and HtrA4 by primary and BeWo trophoblasts. Significance was defined as *p < 0.05, **P < 0.01, ***P < 0.001, ****P < 0.0001.

## Results

### HtrA4 expression increases during spontaneous syncytialization of primary human trophoblasts and significantly correlates to β-hCG

**Figure 1:**
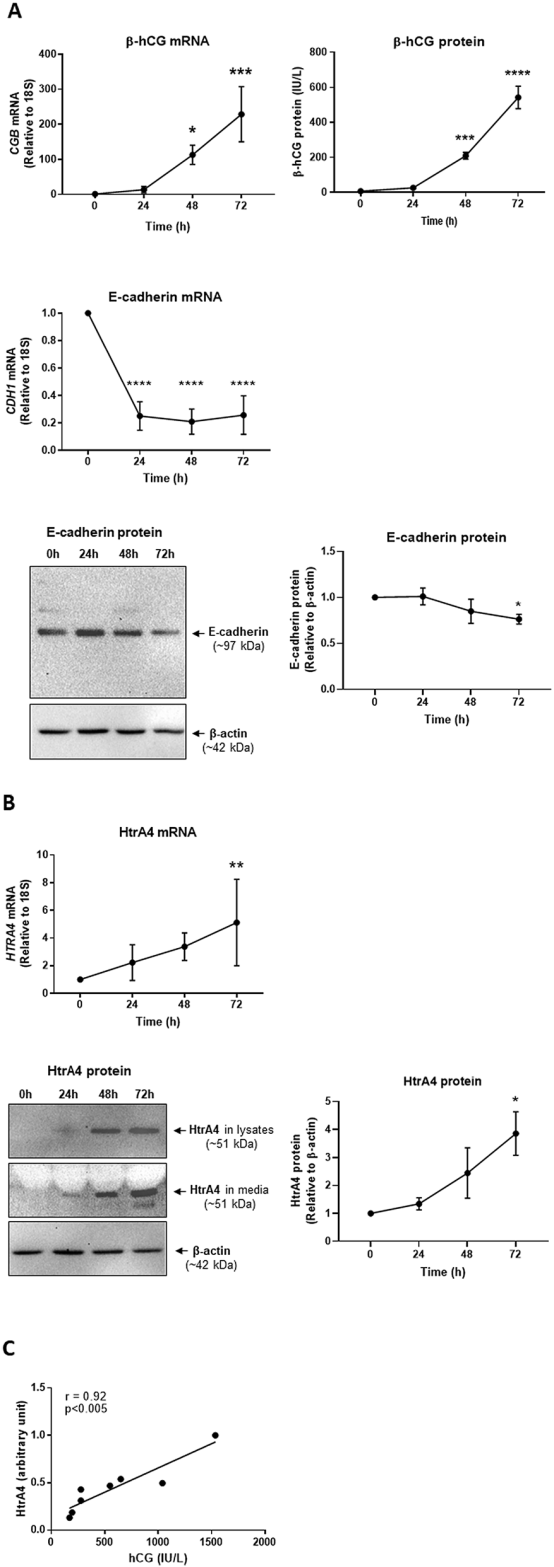
HtrA4 expression increases during syncytialization of primary human trophoblasts. Term placental cytotrophoblasts were cultured for 0, 24, 48 or 72 h, and analysed by Real-time RT-PCR, ELISA and Western blot. Quantitative data are expressed as mean ± SD, n = 4 (unless specified below). For Western blot, a representative image is shown with β-actin as a loading control. (**A**) Confirmation of spontaneous syncytialization. Analysis of β-hCG (*CGB*) and E-cadherin (*CDH1*) mRNA and protein (β-hCG by ELISA, E-cadherin by Western blot). The band intensity of E-cadherin was determined by densitometric analysis and normalised to β-actin, n = 3. Full-length gels are presented in Supplementary Fig. 1A-B. **(B**) HtrA4 increases during syncytialization. Analysis of HtrA4 mRNA and protein. Western blots of both lysates (n = 3) and conditioned media (n = 4) are shown. The band intensity of HtrA4 in lysates was determined by densitometric analysis and normalised to β-actin. Full-length gels are presented in Supplementary Fig. 1C-E. (**C**) Correlation between the secreted levels of hCG and HtrA4 in conditioned media. HtrA4 values (arbitrary units, Y-axis) were from densitometric analysis of Western blots and β-hCG (IU/L, X-axis) concentrations were from ELISA (data of 48 h and 72 h for both, n = 4 each time point). *P < 0.05, **P < 0.01, ***P < 0.001, ****P < 0.0001. In (**A**) and (**B**) all values were statistically compared to 0 h. Figure was prepared using Graphpad Prism v9.0.0 (https://www.graphpad.com/scientific-software/prism/), ImageJ v1.8.0_172 (https://www.imagej.nih.gove/ij/download.html) and Microsoft Powerpoint v2012 (Build 13530.20316, https://www.microsoft.com/en-gb/microsoft-365/powerpoint).

Primary human cytotrophoblasts isolated from healthy term placentas were cultured up to 72 h, and the mRNA and protein levels of syncytial markers β-hCG and E-cadherin were determined every 24 h to confirm spontaneous syncytialization. The β-hCG (*CGB*) mRNA, assessed by real-time RT-PCR, increased 14-fold by 24 h, 113-fold by 48 h (*p* < 0.05) and 228-fold by 72 h (*p* < 0.001) relative to the cells at 0 h. Concurrently, β-hCG protein in the conditioned media, determined by ELISA, increased progressively over time, rising 4-, 30- (*p* < 0.001) and 77-fold (*p* < 0.0001) by 24, 48, and 72 h respectively (Fig. 1A). In contrast, E-cadherin (*CDH1*) mRNA decreased 80% by 24 h, then remained low for the remainder of the culture period (*p* < 0.0001) (Fig. 1A). Western blot analysis of lysates detected E-cadherin at an expected size of ~ 97 kDa, and densitometric analysis demonstrated that the levels reduced gradually over time (Fig. 1A). These data confirmed that primary human trophoblasts under our culture condition spontaneously syncytialized as previously reported^11,29,30^.

HtrA4 mRNA and protein in these cells were then examined by real-time RT-PCR and Western blot analysis. HtrA4 mRNA increased progressively over time, increasing 2-fold by 24 h, 3-fold by 48 h, and 5-fold by 72 h (*p* < 0.01) (Fig. 1B). HtrA4 protein likewise increased in cell lysates as well as in the conditioned media, and a band at expected size of ~ 51 kDa was detected clearly from 48 h onwards (Fig. 1B). Densitometric analysis showed that the levels of HtrA4 in cell lysates increased 140% by 48 h and 286% by 72 h (*p* < 0.05) (Fig. 1B). These results demonstrated that HtrA4 is up-regulated in primary trophoblasts during spontaneous syncytialization.

Next, the correlation between HtrA4 and β-hCG levels in the conditioned media at 48 h and 72 h was analyzed by Pearson’s correlation. HtrA4 levels were determined by densitometric analysis of Western blots and β-hCG by ELISA. The two secreted proteins showed a highly significant (*p* < 0.005) and positive (r = 0.92) correlation (Fig. 1C), suggesting that the up-regulation of HtrA4 and β-hCG is tightly linked during primary trophoblast syncytialization.

### HtrA4 expression also increases during forskolin-induced BeWo syncytialization and significantly correlates to β-hCG

**Figure 2:**
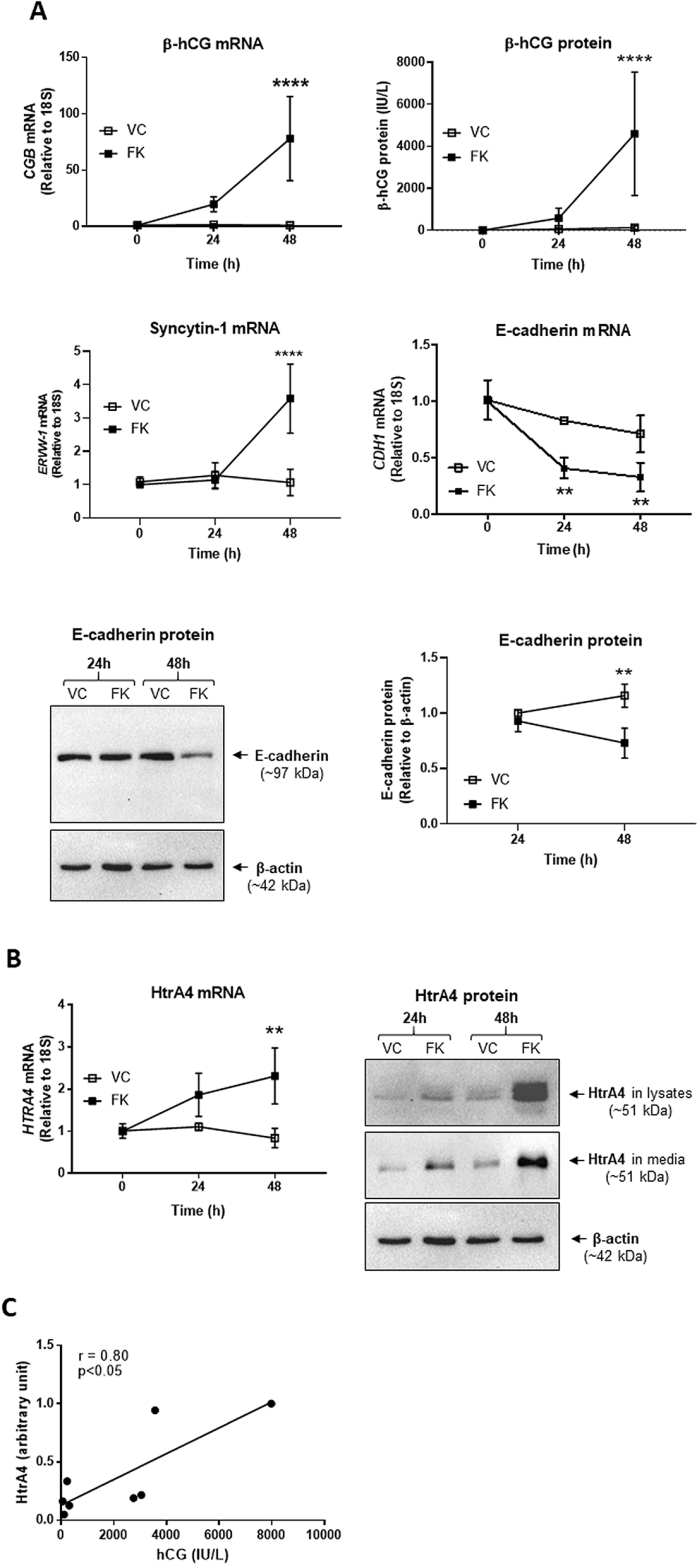
HtrA4 expression increases during BeWo syncytialization. BeWo cells were treated with vehicle control (VC) or 20 µM forskolin (FK) for 0, 24 or 48 h, and analysed by Real-time RT-PCR, ELISA and Western blot. Quantitative data are expressed as mean ± SD, n = 4 (unless specified below). For Western blot, a representative image is shown with β-actin as a loading control. (**A**) Confirmation of forskolin-induced syncytialization. Analysis of β-hCG (*CGB*), syncytin-1 (*ERVW-1*), and E-cadherin (*CDH1*) mRNA, and β-hCG and E-cadherin proteins (β-hCG by ELISA, E-cadherin by Western blot). The band intensity of E-cadherin was determined by densitometric analysis and normalised to β-actin, n = 3. Full-length gels are presented in Supplementary Fig. 2A–B. (**B**) HtrA4 expression increases during forskolin-induced syncytialization. Analysis of HtrA4 mRNA and protein. Western blots of both lysates and conditioned media are shown. Full-length gels are presented in Supplementary Fig. 2C-E. (**C**) Correlation between the levels of secreted hCG and HtrA4 in forskolin-treated conditioned media. HtrA4 values (arbitrary units, Y-axis) were from densitometric analysis of Western blots and β-hCG (IU/L, X-axis) concentrations were from ELISA (data of 48 h and 72 h for both, n = 4 for each time point). **P < 0.01, ****P < 0.0001. Statistical analyses in (**A**) and (**B**) were between VC and FK for each time point. Figure was prepared using Graphpad Prism v9.0.0 (https://www.graphpad.com/scientific-software/prism/), ImageJ v1.8.0_172 (https://www.imagej.nih.gove/ij/download.html) and Microsoft Powerpoint v2012 (Build 13530.20316, https://www.microsoft.com/en-gb/microsoft-365/powerpoint).

We next examined BeWo cell line, which exhibits low levels of spontaneous syncytialization, but can be stimulated by forskolin to accelerate cell–cell fusion. BeWo cells were cultured without and with forskolin for up to 48 h, and the mRNA and protein levels of β-hCG and E-cadherin were analyzed at 24 h and 48 h. The β-hCG (*CGB*) mRNA increased significantly only in cells treated with forskolin, and the levels rose 19-fold by 24 h and 78-fold by 48 h (*p* < 0.0001) compared to vehicle control (Fig. 2A). Similarly, β-hCG protein in the conditioned media of cells treated with forskolin increased tenfold by 24 h and 35-fold by 48 h (*p* < 0.01) compared to the vehicle control (Fig. 2A). Furthermore, syncytin-1 mRNA increased significantly (*p* < 0.0001) only in cells treated with forskolin (Fig. 2A). Conversely, E-cadherin (*CDH1*) mRNA decreased significantly (*p* < 0.01) over time following forskolin treatment compared to the vehicle control (Fig. 2A). Western blot and densitometric analysis showed a clear reduction in E-cadherin protein in lysates following 48 h of forskolin stimulation (*p* < 0.01) (Fig. 2A). These data collectively confirmed that forskolin induced syncytialization in BeWo cells.

HtrA4 mRNA and protein were then examined in above BeWo cells by real-time RT-PCR analysis and Western blot. HtrA4 mRNA increased gradually upon forskolin treatment, rising 1.7-fold by 24 h and 2.9-fold (*p* < 0.01) by 48 h relative to the vehicle control (Fig. 2B). HtrA4 protein in lysates was more clearly detected in cells treated with forskolin, and the levels were much higher at 48 h than 24 h (Fig. 2B). HtrA4 protein was also detected in the conditioned media at 48 h, and the levels were greater in cells treated with forskolin than vehicle control (Fig. 2B). HtrA4 protein in BeWo was also detected at ~ 51 kDa as seen in primary cells, however, a doublet of bands displayed in BeWo whereas a single band was detected in primary cells (Figs. 1B and 2B), suggesting that HtrA4 protein in BeWo may be further post-translationally modified. Nevertheless, these results demonstrated that HtrA4 is up-regulated in BeWo during forskolin-induced syncytialization.

Next, the correlation between HtrA4 and β-hCG levels in the conditioned media at 48 h and 72 h was analyzed by Pearson’s correlation. HtrA4 levels were determined by densitometric analysis of Western blots and β-hCG by ELISA. The two factors again displayed a significant (*p* < 0.05) and positive (r = 0.80) correlation (Fig. 2C), mirroring the findings in primary cytotrophoblasts (Fig. 1C). These data suggest that HtrA4 and β-hCG are also tightly linked during forskolin-induced syncytialization of BeWo cells.

### Stable knockout of HtrA4 in BeWo cells

**Figure 3:**
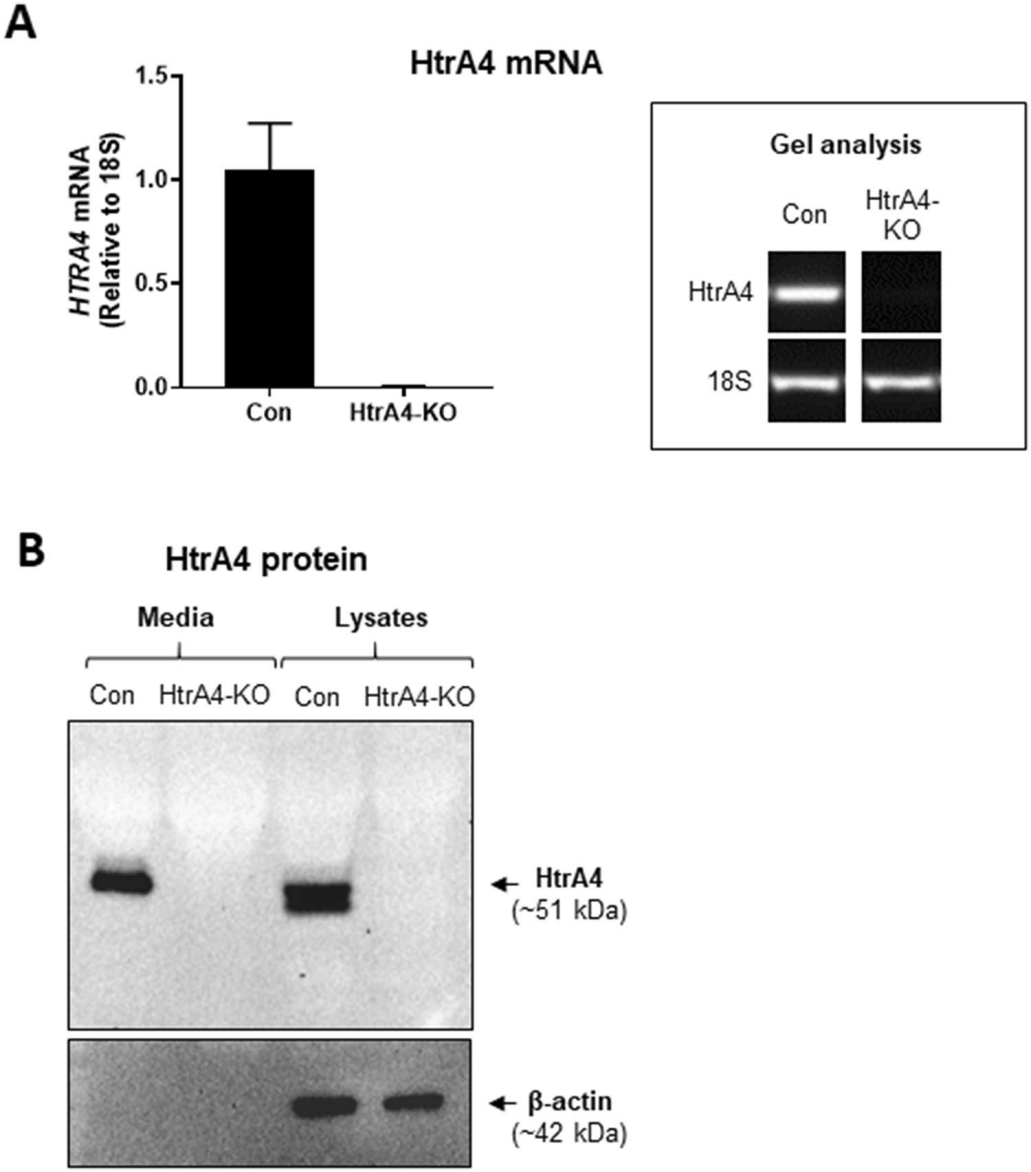
Confirmation of HtrA4 knockout in BeWo cells. (**A**) Analysis of HtrA4 mRNA. Real-time RT-PCR and agarose gel analysis of HtrA4 mRNA in control (Con) and HtrA4-knockout (HtrA4-KO) cells. Data are expressed as mean ± SD, n = 3. A representative gel analysis of PCR products is shown. 18S was used as a loading control. Full-length gels are presented in Supplementary Fig. 3A. (**B**) Analysis of HtrA4 protein. Representative Western blots of HtrA4 in the conditioned medium and lysates are shown, β-actin used as a loading control. Full-length gels are presented in Supplementary Fig. 3B-C. Figure was prepared using Graphpad Prism v9.0.0 (https://www.graphpad.com/scientific-software/prism/) and Microsoft Powerpoint v2012 (Build 13530.20316, https://www.microsoft.com/en-gb/microsoft-365/powerpoint).

As primary cytotrophoblasts do not proliferate in culture, stable knockout of HtrA4 was not performed in these cells because it requires repeated passaging. Instead, BeWo cells were chosen to further investigate the importance of HtrA4 in trophoblast syncytialization. The HtrA4 gene in BeWo was stably knocked out by CRISPR/Cas9 double nickase, and the knockout was confirmed by RT-PCR and Western blot analysis. HtrA4 mRNA was readily detected in control transfected BeWo, but undetectable in HtrA4-knockout (HtrA4-KO) cells (Fig. 3A). Agarose gel analysis of RT-PCR products displayed the expected band of 168 bp in BeWo of control transfection but not in HtrA4-KO cells (Fig. 3A). HtrA4 protein was examined by Western blot analysis in conditioned media as well as in cell lysates following 48 h culture. HtrA4 was detected only in the control but not in HtrA4-KO cells (Fig. 3B). This was validated with three different HtrA4 antibodies (data not shown). These results confirmed that a stable HtrA4-KO BeWo cell line was successfully created.

### HtrA4-knockout BeWo cells fail to increase β-hCG or syncytin-1, or down-regulate E-cadherin upon forskolin stimulation

**Figure 4:**
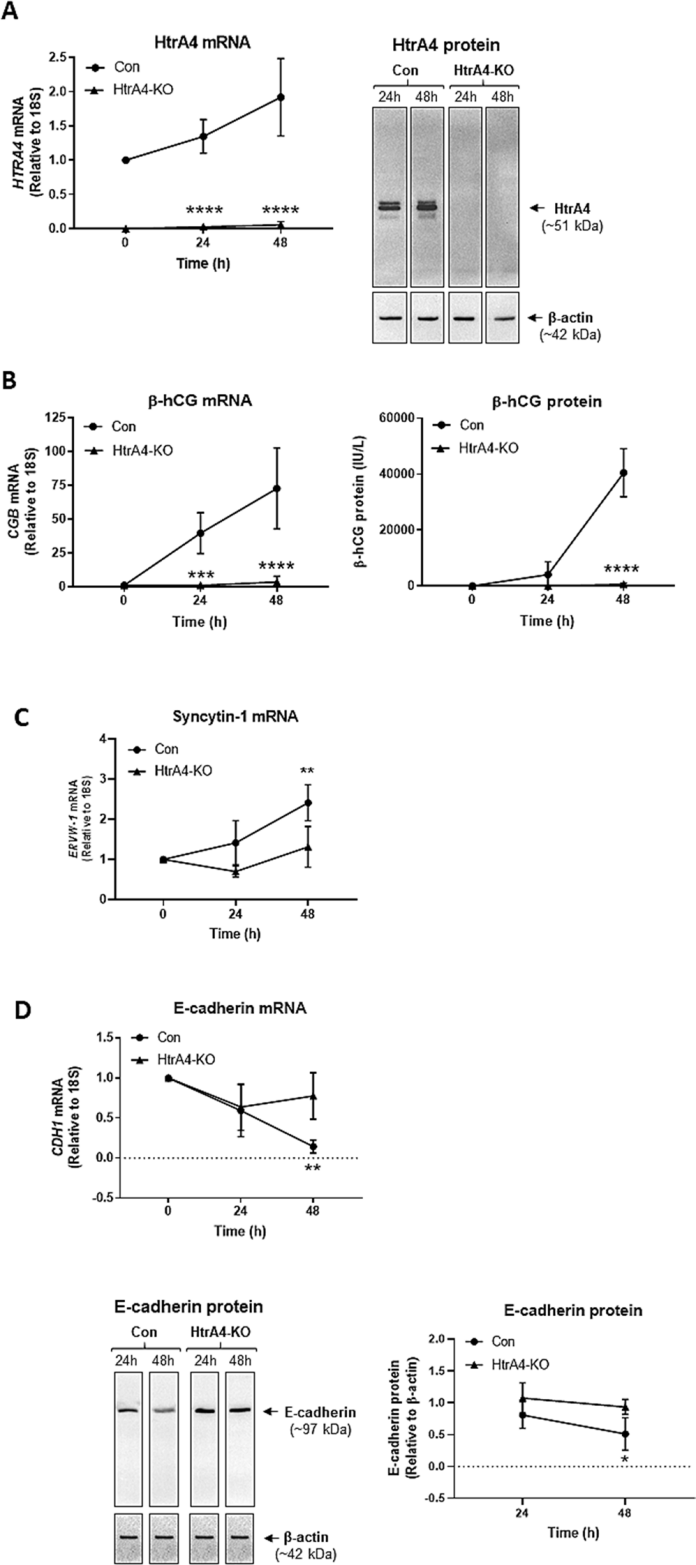
BeWo cells without HtrA4 fail to increase β-hCG or syncytin-1 or down-regulate E-cadherin upon forskolin stimulation. Control (Con) and HtrA4-knockout (HtrA4-KO) BeWo cells were treated with 20 µM forskolin for 0, 24 or 48 h, and analysed by real-time RT-PCR, ELISA and Western blot. Quantitative data are expressed as mean ± SD, n = 3–4. For Western blot, a representative image is shown with β-actin as a loading control. (**A**) HtrA4 mRNA and protein. Western blot of lysate is shown. Full-length gels are presented in Supplementary Fig. 4A. (**B**) β-hCG (*CGB*) mRNA and protein (β-hCG by ELISA). (**C**) Syncytin-1 (*ERVW-1)* mRNA. (**D**) E-cadherin (*CDH1*) mRNA and protein (Western blot). The band intensity of E-cadherin was determined by densitometric analysis and normalised to β-actin, n = 4. Full-length gels are presented in Supplementary Fig. 4B-C. *P < 0.05, **P < 0.01, ***P < 0.001, ****P < 0.0001. Statistical analyses were between Con and HtrA4-KO for each time point. Figure was prepared using Graphpad Prism v9.0.0 (https://www.graphpad.com/scientific-software/prism/), ImageJ v1.8.0_172 (https://www.imagej.nih.gove/ij/download.html) and Microsoft Powerpoint v2012 (Build 13530.20316, https://www.microsoft.com/en-gb/microsoft-365/powerpoint).

Control and HtrA4-KO BeWo cells were treated with forskolin for up to 48 h, and HtrA4 and syncytial markers was assessed at 24 and 48 h (Fig. 4). Following forskolin treatment, control cells (transfected with control plasmids) significantly increased HtrA4 mRNA as expected (*p* < 0.0001 at 24 h, and *p* < 0.0001 at 48 h), whereas no HtrA4 expression was detected in HtrA4-KO cells at any time point (Fig. 4A). HtrA4 protein in the media (data not shown) and lysates (Fig. 4A) was also analyzed by Western blot. HtrA4 was detected in the control with the levels increasing over time, but it was completely absent in the HtrA4-KO cells (Fig. 4A). These data confirmed that HtrA4 was absent in HtrA4-KO BeWo cells, and could not be up-regulated by forskolin.

We next examined changes in syncytial markers in above control and HtrA4-KO cells. Over the 48 h period following forskolin treatment, β-hCG mRNA increased considerably in control cells as expected, however, it did not change in HtrA4-KO cells and remained significantly lower than the control (*p* < 0.001 at 24 h, and *p* < 0.0001 at 48 h) (Fig. 4B). Similarly, β-hCG protein in the conditioned media increased substantially over time in control cells, but not in HtrA4-KO cells which showed minimal levels and was significantly (*p* < 0.0001) lower than the control at 48 h (Fig. 4B). Likewise, syncytin-1 mRNA increased over time in control cells but not in HtrA4-KO cells, and the levels between the two were significantly different (*p* < 0.01) at 48 h (Fig. 4C). Furthermore, following forskolin treatment, E-cadherin mRNA decreased progressively in control, but not significantly in HtrA4-KO cells, and the difference between the two groups were highly significant (*p* < 0.01) at 48 h (Fig. 4D). Similarly, Western blot and densitometric analysis demonstrated that by 48 h forskolin reduced E-cadherin protein levels significantly (*P* < 0.05) in control but not in HtrA4-KO cells, and the levels were significantly lower in control than HtrA4-KO cells (Fig. 4D).

### Control but not HtrA4-KO BeWo cells increase the formation of multinucleated cells upon forskolin stimulation

**Figure 5:**
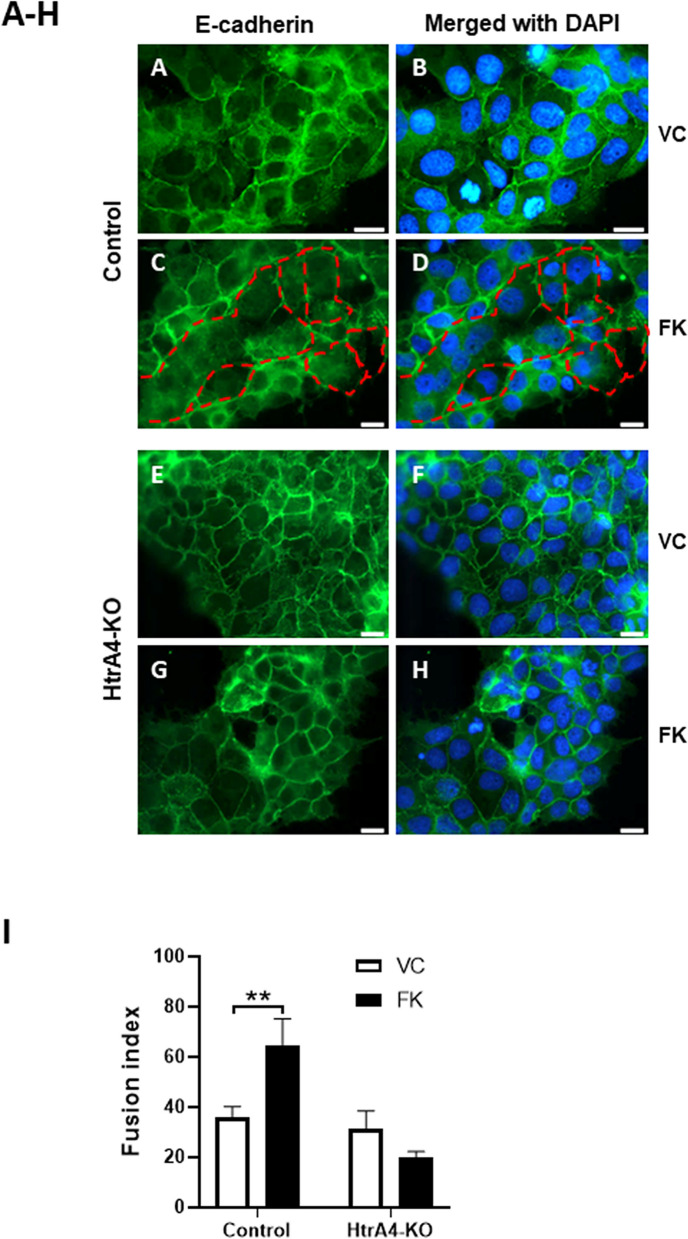
BeWo cells without HtrA4 fail to increase the formation of multinucleated cells. Control and HtrA4-knockout (HtrA4-KO) BeWo cells were treated with vehicle control (VC) or 20 µM forskolin (FK) for 48 h. E-cadherin was examined by immunofluorescence (green), and nuclei were stained with DAPI (blue). (**A**–**H**) Representative images. (**A**–**D**) Control, (**E**–**H**) HtrA4-knockout cells. Scale bars, 500 µm. Dotted red lines outline multinucleated cells. (**I**) Fusion index. Data are expressed as mean ± SD, n = 3. **P < 0.01. Figure was prepared using Graphpad Prism v9.0.0 (https://www.graphpad.com/scientific-software/prism/), Olympus cellSens Standard Imaging Software v1.16 (https://www.olympus-lifescience.com/en/software/cellsens/) and Microsoft Powerpoint v2012 (Build 13530.20316, https://www.microsoft.com/en-gb/microsoft-365/powerpoint).

Control and HtrA4-KO BeWo cells were treated with vehicle control or forskolin for 48 h, and E-cadherin was analyzed by immunofluorescence (Fig. 5). In control cells, when treated with vehicle control (Fig. 5A, B), E-cadherin staining was detected mainly around individual cells; however, when treated with forskolin (Fig. 5C, D), cells in patches lost E-cadherin staining and became multinucleated. In contrast, in HtrA4-KO cells, E-cadherin was detected primarily around individual cells in both vehicle control (Fig. 5E, F) and forskolin (Fig. 5G, H) treated groups, with the two showing no obvious differences (Fig. 5F, H). Cell fusion was further quantified by fusion index analysis. For control cells, the fusion index increased significantly following forskolin treatment compared to vehicle control (*p* < 0.01) (Fig. 5I). However, this was not observed in HtrA4-KO cells, which showed a basal fusion index similar to the control cells, but fusion index did not change significantly upon forskolin stimulation compared to vehicle control (Fig. 5I). These data therefore suggest that the forskolin-induced syncytialization occurred successfully in control BeWo cells, but not at all in HtrA4-KO cells.

## Discussion

In this study we demonstrated for the first time that HtrA4 expression increases during spontaneous syncytialization of primary human cytotrophoblasts as well as during forskolin-induced syncytialization of BeWo cells. To investigate the importance of HtrA4 in syncytialization, we knocked out HtrA4 in BeWo and showed that cells without HtrA4 failed to undergo forskolin-induced syncytialization. Collectively, our data suggest a critical role of HtrA4 in promoting trophoblast syncytialization.

Previous studies localized HtrA4 expression to the cytoplasm of cytotrophoblasts and syncytiotrophoblasts in the human placenta^22,23^, however, it is unknown whether HtrA4 is functionally involved in the process of syncytialization. We have previously reported that BeWo, the only cell line that can efficiently syncytialize in culture when treated with forskolin or cAMP^31–33^, is also the only cell line, among a number of trophoblast cell lines examined, that expresses a high level of HtrA4^21^. Based on this background, in this study we first examined HtrA4 expression in freshly isolated primary human cytotrophoblasts during syncytialization, which occurs spontaneously in culture, and in BeWo cells, which syncytialize upon stimulation with forskolin. The success of syncytialization in each model was confirmed by significant up-regulation and secretion of β-hCG, and by the concurrent down-regulation of E-cadherin. In both cell models, HtrA4 mRNA and protein levels increased concurrently with syncytialization.

As a secreted protein, HtrA4 levels in the conditioned media of both primary trophoblasts and BeWo cells were also elevated during syncytialization, consistent with placenta-derived HtrA4 being detected in the blood circulation of pregnant women^21–23^. Furthermore, a highly significant and positive correlation was found between the secreted levels of β-hCG and HtrA4 in the conditioned media in both cell models. Considering β-hCG is mainly produced and secreted by the syncytiotrophoblast and acts in an autocrine manner to increase syncytium formation^34,35^, our data suggest that HtrA4 may be essential for the expression and secretion of β-hCG and syncytialization. To further investigate this proposition, we next knocked out HtrA4 in BeWo cell line and examined the consequences.

While control-transfected BeWo cells responded to forskolin, profoundly up-regulated β-hCG and syncytin-1, and down-regulated E-cadherin, as observed in the wild type BeWo cells, HtrA4-KO BeWo did not alter these syncytialization markers upon forskolin treatment. Failed syncytialization was also confirmed morphologically. Upon forskolin stimulation, BeWo cells fuse to form multinucleated syncytiotrophoblasts^36–38^, which can be visualized by immunostaining for E-cadherin that outlines individual cell membranes^30,38,39^. Utilizing this strategy, we demonstrated that control BeWo cells, following forskolin treatment, formed many multinucleated cells as expected with a significant elevation in fusion index. In contrast, HtrA4-KO cells did not undergo any distinct changes in morphology or alter fusion index when treated with forskolin. These data provided strong biochemical as well as morphological evidence that HtrA4 plays an important role in syncytialization.

The mechanistic basis of HtrA4 in promoting syncytialization remains to be investigated. Malhotra et al. previously reported that knockdown of β-hCG decreases BeWo syncytialization^12^, our data presented here therefore suggest that HtrA4 may regulate the process of syncytialization pathway upstream of β-hCG. Previous studies have also highlighted the critical importance of E-cadherin reduction in trophoblast differentiation^19,38,40^, our results thus suggest that HtrA4 action occurs upstream of E-cadherin in the syncytialization process. Given the structural similarities of HtrA4 to HtrA1 and HtrA3, HtrA4 may share some functions of these two family members. Both HtrA1 and HtrA3 are known to bind and cleave several extracellular matrix (ECM) proteins to regulate ECM remodelling^41–46^. In the placental villi, a host of ECM molecules play an essential role in supporting cell growth, tissue remodelling, and villous architecture^47^. Therefore, it can be speculated that HtrA4 may cleave cell surface receptors and ECM components to promote syncytialization, and deletion of HtrA4 may consequently inhibits the morphological changes required for cell fusion.

Our results contradict the findings of Wang *et al*^48^. While our findings demonstrated that HtrA4 expression promotes syncytialization, Wang et al. reported that increased HtrA4 expression suppresses cell fusion^48^. A major reason for this discrepancy lies in the difference of cell model and experimental approach used. Wang et al. investigated the effect of HtrA4 on cell fusion using HEK293T cells^48^, which is a human embryonic kidney-derived cell line. HEK293T cells do not represent trophoblasts, do not naturally syncytialize or express any established syncytialization markers. Importantly, HEK293T cells do not naturally express HtrA4. Given the vast differences in study design, the conclusions published by Wang et al. cannot be compared to the findings of this study. However, our findings presented here strongly support a critical and promoting role of HtrA4 in trophoblast syncytialization, evidenced not only by significant up-regulation of HtrA4 during syncytialization of primary human trophoblasts as well as trophoblast BeWo cell line, but also by the clear failure of BeWo cells to syncytialize when HtrA4 was knocked out.

One limitation of our study is that BeWo cell line was used to knock out HtrA4. While BeWo is a well-established trophoblast cell line to study syncytialization, it does not reflect all the features of the primary villous cytotrophoblast^49^. However, primary trophoblasts do not proliferate well in culture and it is impractical to use them to perform stable gene knockout studies. Nevertheless, since HtrA4 regulation during BeWo syncytialization strongly resembles that of primary trophoblasts, we believe the data obtained from BeWo provide convincing evidence for a critical role of HtrA4 in promoting trophoblast syncytialization in general.

In summary, we demonstrated that HtrA4 expression is significantly increased during syncytialization of both primary human cytotrophoblasts and BeWo cell line. We further showed that the loss of HtrA4 gene expression prevented BeWo cells from syncytializing. These data collectively suggest that HtrA4 plays an essential role in syncytialization.

## Supplementary Information

Supplementary Information.
