# Supplementary material for: HtrA4 is up-regulated during trophoblast syncytialization and BeWo cells fail to syncytialize without HtrA4
Source: Sci Rep. 2021 Jul 13;11:14363. doi: 10.1038/s41598-021-93520-1 (PMC8277827; doi:10.1038/s41598-021-93520-1)
Supplement: Supplementary file 1 — Supplementary Information. [file 41598_2021_93520_MOESM1_ESM.pdf]

HtrA4 is up-regulated during trophoblast syncytialization and BeWo cells fail to syncytialize without HtrA4

Mary Mansilla[1,2,3], Yao Wang[1,2,3], Rebecca Lim[4,5], Kirsten Palmer[4] and Guiying Nie[1,2,3],*

[1]Implantation and Placental Development Laboratory, Centre for Reproductive Health, Hudson Institute of Medical Research, Clayton, Victoria, 3168, Australia;

[2]Department of Molecular and Translational Science, Monash University, Clayton, Victoria, 3168, Australia;

[3]Implantation and Pregnancy Research Laboratory, School of Health and Biomedical Sciences, RMIT University, Bundoora, Victoria, 3083, Australia.

[4]Department of Obstetrics and Gynaecology, Monash University, Clayton, Victoria, 3168, Australia;

[5]Australian Regenerative Medicine Institute, Monash University, Clayton, Victoria, 3800, Australia;

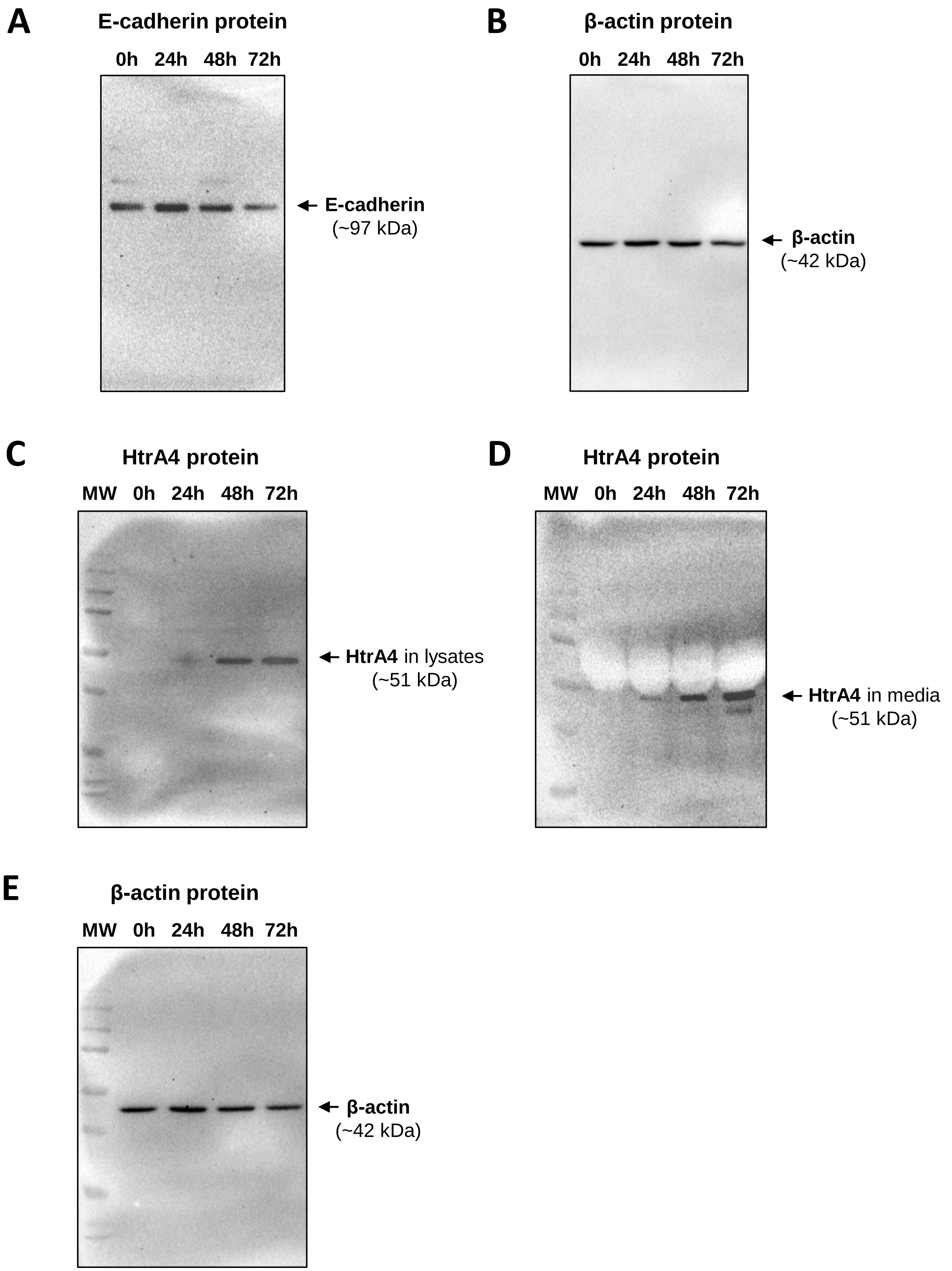

Supplementary Figure 1. Full Western blot images for Figure 1. (**A-B**) Full Western blot images for Figure 1A. (**A**) E-cadherin and (**B**) β-actin. (**C-E**) Full Western blot images for Figure 1B. (**C**) HtrA4 in lysates, (**D**) HtrA4 in conditioned media and (**E**) β-actin. MW = molecular weight. Figure was prepared using Microsoft Powerpoint v2012 (Build 13530.20316, https://www.microsoft.com/en-gb/microsoft-365/powerpoint).

Supplementary Figure 1

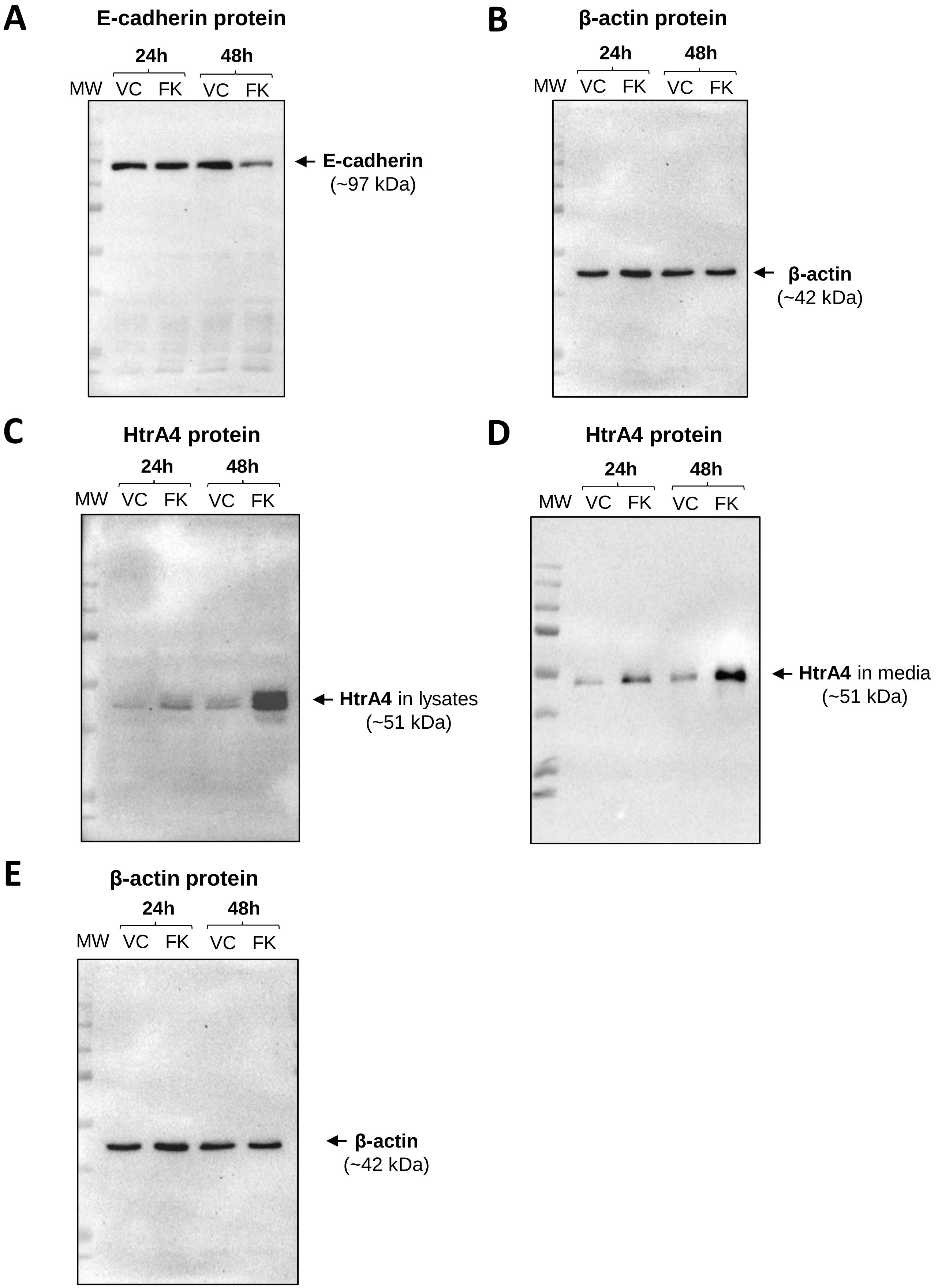

Supplementary Figure 2. Full Western blot images for Figure 2. (**A-B**) Full Western blot images for Figure 2A. (**A**) E-cadherin and (**B**) β-actin. (**C-E**) Full Western blot images for Figure 2B. (**C**) HtrA4 in lysates, (**D**) HtrA4 in conditioned media and (**E**) β-actin. MW = molecular weight, VC = vehicle control, FK = forskolin. Figure was prepared using Microsoft Powerpoint v2012 (Build 13530.20316, https://www.microsoft.com/en-gb/microsoft-365/powerpoint).

Supplementary Figure 2

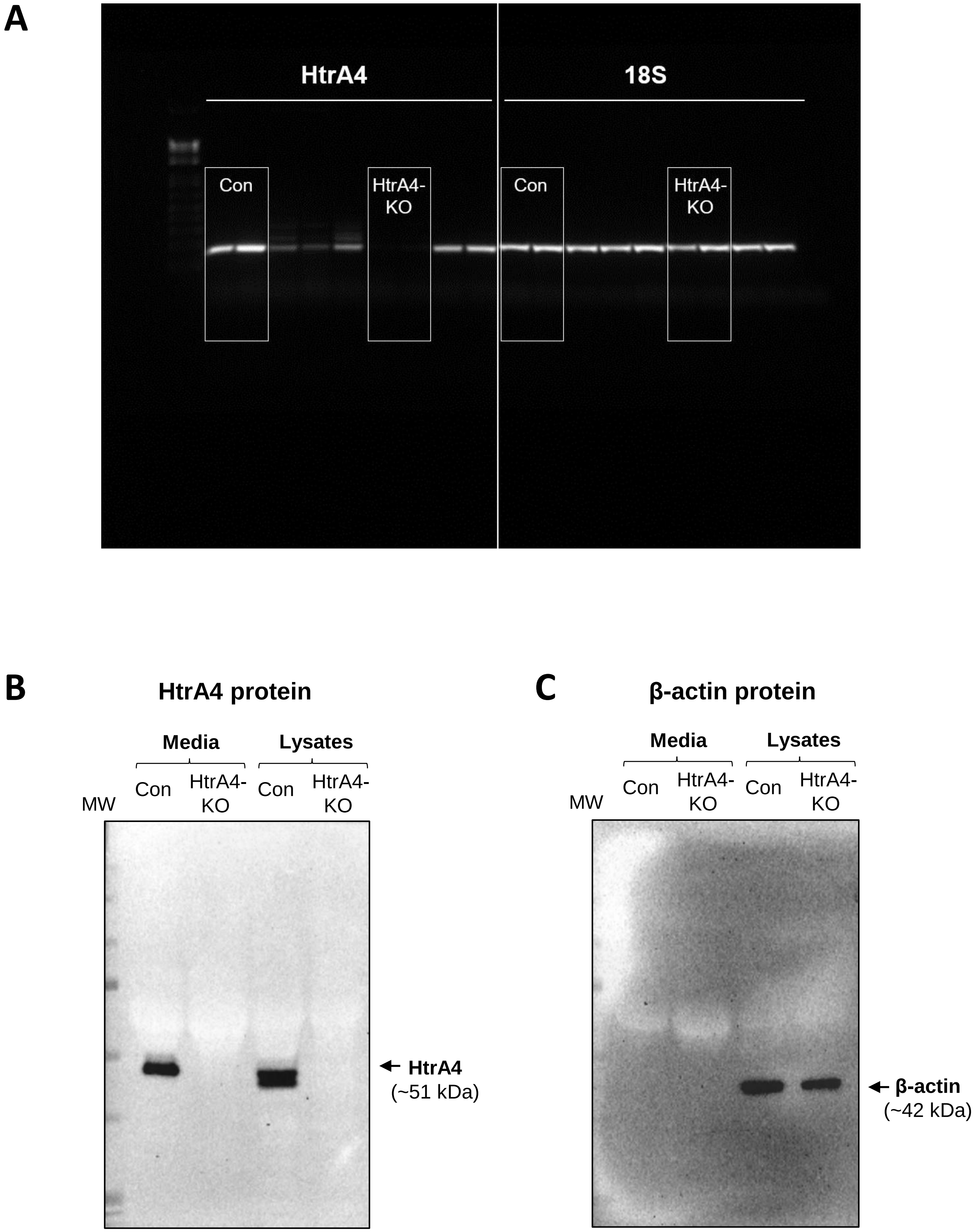

Supplementary Figure 3. Full images of Figure 3. (**A**) Full agarose gel images of RT-PCR analysis of HtrA4 mRNA and 18S in control (Con) and HtrA4-knockout (HtrA4-KO) cells shown in Figure 3A. (**B-C**) Full Western blot images for Figure 3B. (**B**) HtrA4 and (**C**) β-actin. MW = molecular weight**.**. Figure was prepared using Microsoft Powerpoint v2012 (Build 13530.20316, https://www.microsoft.com/en-gb/microsoft-365/powerpoint).

Supplementary Figure 3

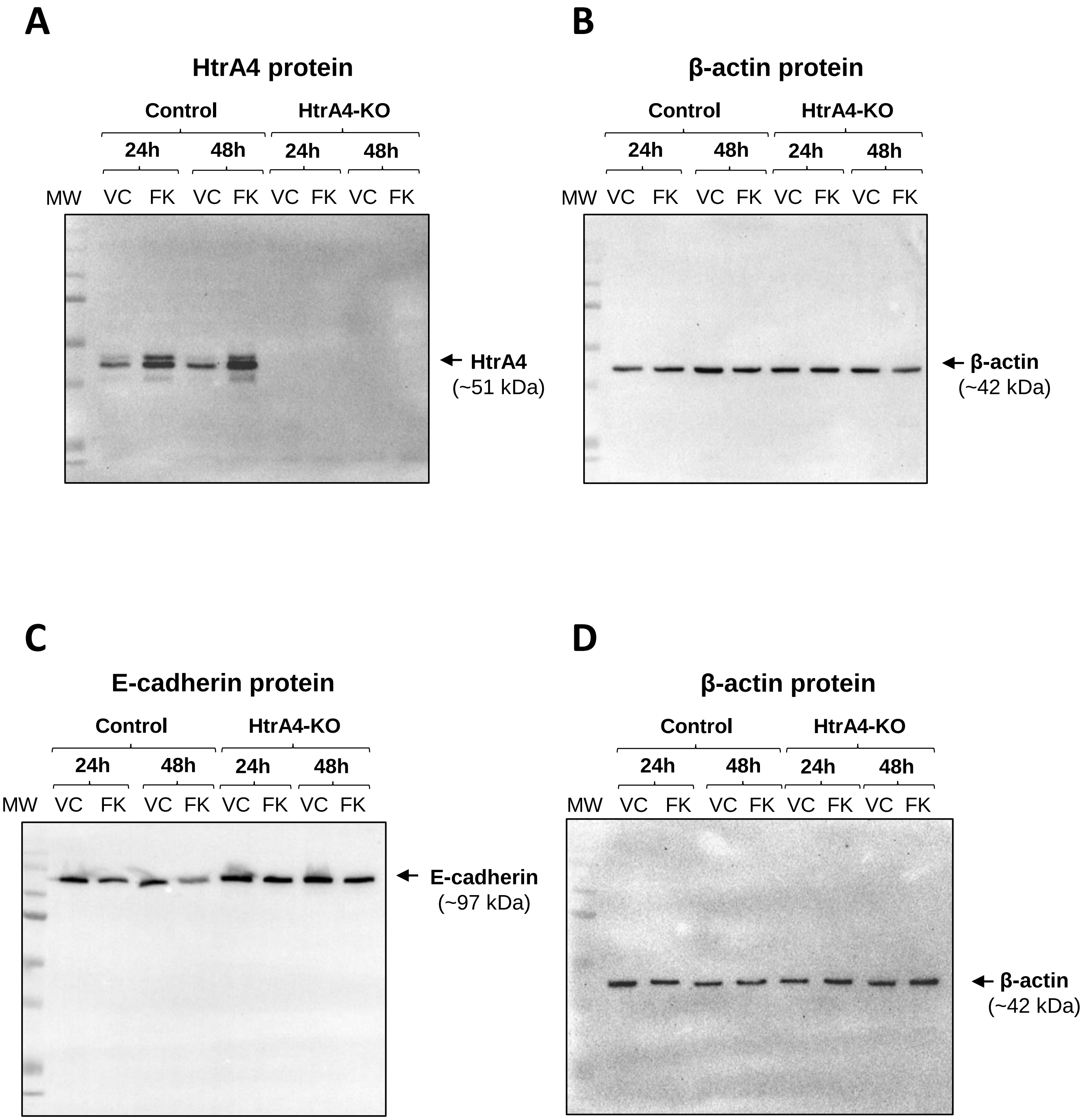

Supplementary Figure 4. Full Western blot images of Figure 4. (**A-B**) Full Western blot images for Figure 4A. (**A**) HtrA4 and (**B**) β-actin. (**C-D**) Full Western blot images for Figure 4D. (**C**) E=cadherin and (**D**) β-actin. HtrA4-KO = HtrA4 knockout, MW = molecular weight, VC = vehicle control, FK = forskolin. Please note VC data were not presented in Figures 4A or 4D in the manuscript. Figures were prepared using Microsoft Powerpoint v2012 (Build 13530.20316, https://www.microsoft.com/en-gb/microsoft-365/powerpoint).

Supplementary Figure 4
